# Candidate loci for leaf angle in maize revealed by a combination of genome-wide association study and meta-analysis

**DOI:** 10.3389/fgene.2022.1004211

**Published:** 2022-11-11

**Authors:** Haiyang Duan, Jianxin Li, Yan Sun, Xuehang Xiong, Li Sun, Wenlong Li, Jionghao Gao, Na Li, Junli Zhang, Jiangkuan Cui, Zhiyuan Fu, Xuehai Zhang, Jihua Tang

**Affiliations:** ^1^ National Key Laboratory of Wheat and Maize Crop Science, College of Agronomy, Henan Agricultural University, Zhengzhou, China; ^2^ State Key Laboratory of Crop Stress Adaptation and Improvement, School of Life Sciences, Henan University, Kaifeng, China; ^3^ College of Plant Protection, Henan Agricultural University, Zhengzhou, China; ^4^ The Shennong Laboratory, Zhengzhou, China

**Keywords:** maize, leaf angle, genome-wide association study, meta-analysis, ideal plant architecture

## Abstract

Leaf angle (LA) is a key component of maize plant architecture that can simultaneously govern planting density and improve final yield. However, the genetic mechanisms underlying LA have not been fully addressed. To broaden our understanding of its genetic basis, we scored three LA-related traits on upper, middle, and low leaves of 492 maize inbred lines in five environments. Phenotypic data revealed that the three LA-related traits were normally distributed, and significant variation was observed among environments and genotypes. A genome-wide association study (GWAS) was then performed to dissect the genetic factors that control natural variation in maize LA. In total, 85 significant SNPs (involving 32 non-redundant QTLs) were detected (*p* ≤ 2.04 × 10^–6^), and individual QTL explained 4.80%–24.09% of the phenotypic variation. Five co-located QTL were detected in at least two environments, and two QTLs were co-located with multiple LA-related traits. Forty-seven meta-QTLs were identified based on meta-analysis combing 294 LA-related QTLs extracted from 18 previously published studies, 816 genes were identified within these meta-QTLs, and seven co-located QTLs were jointly identified by both GWAS and meta-analysis. *ZmULA1* was located in one of the co-located QTLs, *qLA7*, and its haplotypes, hap1 and hap2, differed significantly in LA-related traits. Interestingly, the temperate materials with hap2 had smallest LA. Finally, we also performed haplotype analysis using the reported genes that regulate LA, and identified a lot of maize germplasms that aggregated favorable haplotypes. These results will be helpful for elucidating the genetic basis of LA and breeding new maize varieties with ideal plant architecture.

## Introduction

Leaf angle (LA) is one of the key traits of maize plant architecture. Upright leaves can make maize plants more compact, enable them to adapt to high planting density, and reduce effects of shading between plants, thereby increasing photosynthetic efficiency and final grain yield (Stewart et al., 2003). Maize yield in the United States has increased eight-fold in the past 90 years. During this period, changes in maize LA have altered plant architecture, resulting in new varieties that are tolerant of high planting densities, allowing for more efficient light capture as planting density has increased (Tian et al., 2011). Compact plant architecture with upright leaves can increase the ability of crops to capture light energy and increase final yield (Saitoh et al., 2002).

LA is one of the important indicators used to describe maize canopy structure (Arkebauer et al., 2009) and is a quantitative trait. Thirty QTLs related to upper leaf angle were identified in a Nested Association Mapping Population (NAM) composed of 25 recombinant inbred line (RIL) families, including 4,892 lines (Tian et al., 2011). A maize BC_2_S_3_ population was constructed from teosinte and the maize inbred line W22, 12 QTLs underlying LA were identified. *UPA1*-NIL^W22^ (Near Isogenic Line) has smaller LA (Upper, Middle and Low LA) than *UPA1*-NIL^8759^. On the contrary, *UPA2*-NIL^W22^ has larger LA (Upper, Middle and Low LA) than *UPA2*-NIL^8759^. These results indicated that *UPA1* and *UPA2* have opposite regulatory patterns, and with the increase of planting density, *UPA2*-NIL^8759^ has higher grain yield (kg/ha) than *UPA2*-NIL^W22^ (Tian et al., 2019). Several genes within the quantitative trait loci (QTL) that govern LA have been cloned. *ZmILI1* was located within the *qLA2*, which was identified in a maize F_2:3_ population constructed from the compact inbred line Yu82 and the expanded inbred line Yu87-1. *ZmILI1* (*Zm00001d002121*) binds to the promoter of *CYP90D1* (*Zm00001d039453*) and inhibits expression of *CYP90D1* through a *CYP90D1*-mediated cytochrome P450-catalyzed reaction, Brassinosteroids (BRs) can be delivered to the BR receptor *BAK1*, which binds to the nuclear transcription factors *BZR1*, *BZR2* and *BES1*, resulting in a smaller LA (Upper LA) (Tang et al., 2010; Ren et al., 2020). Li et al. found that the rice gene *oslazy1* regulates LA by affecting gravitropism (Li et al., 2007), its maize homolog *ZmCLA4* regulates LA by changing mRNA accumulation, leading to changes in gravitational properties and cell development (Zhang et al., 2014). Gao et al. used a *Gmilpa1* mutant with increased soybean petiole angle and isolated the gene *GmILPA1*, which encodes the *APC8* protein. It is expressed in leaf primordium cells and can increase petiole angle by promoting the growth and division of leaf occipital cells (Gao et al., 2017).

Genome-wide association study (GWAS) has been widely used in plant genetics research and has proven to be a powerful tool for mining QTLs or causal genes for complex quantitative traits. The first GWAS report in plants was published in 2008. Single nucleotide polymorphism (SNP) haplotypes at 8,590 loci across 10 maize chromosomes were tested for association with kernel oleic acid content in 553 maize inbred lines, and a putative gene responsible for the target trait (*fad2*) was identified (Belo et al., 2008). Recently, Wang et al. performed whole-genome sequencing on 350 elite maize inbred lines representing multiple eras of germplasms from China and the United States and measured 15 agronomic traits for GWAS. A number of key candidate genes, such as *ZmNAC16*, *ZmSBP18*, *ZmPIF4*, and *ZmPIF3.3*, that regulate maize density tolerance and ideal plant architecture were cloned, providing an important foundation for future genomics-enabled maize breeding (Wang et al., 2020). Besides, meta-analysis is an objective method for statistically re-analyzing existing empirical literature, enabling a more unbiased evaluation of the evidence than that provided by traditional narrative commentary (Egger et al., 1997). It has been widely used to summarize and further explore complex biological mechanisms (Makinde et al., 2021), and it has also been applied in genetic studies of crop heterosis, grain yield, and stress tolerance (Li et al., 2011; Thiemann et al., 2014; Sharma et al., 2018; Wang et al., 2021). As an important plant architectural trait, LA affects the ability of the maize canopy to capture light and the light energy utilization efficiency of the population, understanding natural variation in LA and identifying its key genes are very important for breeding maize with high photosynthetic efficiency (Tao et al., 2002).

Here, 492 diverse maize inbred lines were used to investigate LA-related traits at different leaf positions in multiple environments. A GWAS was performed with 1.25 M SNPs to explore natural allelic variations that influence LA; in addition, a large amount of LA QTL data from previously published studies was used for meta-analysis. The main purposes were: 1) to explore the phenotypic variation of LA 2) to identify natural variation of SNPs/loci and candidate genes significantly associated with LA 3) to select germplasms with the favorable haplotypes of LA to improve maize plant architecture. The results will enrich our understanding of the genetic basis of LA and enhance ideotype-based maize breeding.

## Materials and methods

### Plant materials and growth conditions

The association mapping panel (AMP) used in this study consists of 492 maize inbred lines, including 225 tropical/subtropical germplasms and 267 temperate germplasms, which is a subset of 527 inbred lines (Yang et al., 2011). In 2020, all 492 maize inbred lines were planted in Yuanyang Modern Agricultural Science and Technology Park of Henan Agricultural University (Yuanyang, N35°N, E113°E) in the end of April (defined as YYC) and in early July (defined as YYX). In addition, 269 inbreds (73 tropical/subtropical and 196 temperate) were randomly selected from the AMP and planted at the XunXian Experimental Station of Hebi Academy of Agricultural Sciences in Henan province (Hebi, N35°N, E114°E, defined as HB), the Cotton Seed Farm in Yongcheng, Henan (YongCheng, N33°, E116°, defined as YC) and the Yuanyang Modern Agricultural Science and Technology Park of Henan Agricultural University (Yuanyang, N35°, E113°, defined as YY) in early June 2020. All inbred lines were planted in a randomized complete block design with two replications, a single row length of 3 m, a row spacing of 0.67 m, and a final planting density of 45,000 plants/ha in all environments.

### Measurement of LA

LA data from the AMP were collected using a digital angle ruler (digital display 360° angle ruler 0–200 mm, Wenzhou Weidu Electronics Co., Ltd.). To be consistent, we investigated the flowering date of each line, and LA were investigated at 15 days after pollination for each line at all environments. Three Leaf Angle (LA-related traits) was scored at three positions: the upper leaf (first leaf below the flag leaf, ULA), the middle leaf (first leaf above the first ear, MLA), and the lower leaf (second leaf below the first ear, LLA). Only ULA was investigated at YYC and YYX, whereas all three LA-related traits were recorded at HB, YC, and YY. The average values of ULA, MLA, and LLA were calculated from five uniformly growing plants in each row. The average of each LA-related traits for the two replications in each environment was calculated. Overall, ULA at all the five environments (YYC, YYX, YY, HB, and YC), MLA, and LLA at the three environments (YY, HB, and YC) were collected. These phenotypic data was used for general statistical analysis, Pearson correlation analysis, two-way ANOVA, broad-sense heritability calculation, Best Linear Unbiased Prediction (BLUP) and GWAS.

### Statistical analyses of LA

General statistical analyses (e.g. mean, variation range, standard deviation, kurtosis, skewness) were finished in SPSS Statistics V17.0 after removal of outliers. For the five values measured for one trait of a genotype, if a value is not within the range of the mean plus or minus 1.5 times of standard deviation, it will be regarded as an outlier. A repeated-measures two-way ANOVA, using the following formula: V = G + E + G × E + e, here, V is total variance, G is variance of genotype, E is environmental variance, G × E is variance of genotype-environment interaction, e is error. It was also performed in SPSS Statistics V17.0. The mixed linear model of the lme4 package in R (version 4.1.1, R Foundation for Statistical Computing, http://www.r-project.org/) was used to calculate the Best Linear Unbiased Prediction (BLUP) value for each trait in the five environments (Eugster et al., 2011). In addition, the Best Linear Unbiased Prediction (BLUP) was calculated for GWAS, which can estimate the random effects (Coram et al., 2017). For MLA and LLA, broad-sense heritability was computed using the following formula: *H* ^*2*^ = [δ_G_^2^ /δ_G_^2^ +(δ_GE_^2^/*n*)+*δ*_e_^2^/(*nr*)], where δ_G_^2^ is the genotypic variance, δ_GE_^2^ is the genotype × environment variance, δ_e_^2^ is the error variance, *r* is the number of replications, and *n* is the number of environments. Considering the unbalanced data for ULA, the harmonic mean (*h*), *h* = (492 + 269)/(492/2 + 269/3), was used to instead of the number of environments (*n*) for calculating the *H* ^*2*^ (Nyquist and Baker, 1991; Schmidt et al., 2019). Pearson correlation coefficients between paired traits were calculated using the corr function in R (version 4.1.1).

### GWAS

The genotype data used in this study was downloaded from the Maizego website (http://www.maizego.org/Resources.html) and have been deposited in the European Variation Archive (EVA) at EMBL-EBI under accession number PRJEB56161 (https://www.ebi.ac.uk/eva/?eva-study=PRJEB56161). This genotype data was inferred from the Illumina MaizeSNP50 array, reduced-representation genome sequencing (genotyping-by-sequencing, GBS), the high density Affymetrix Axiom Maize 600K array (600K), and previous deep RNA-sequencing data of whole kernels at 15 days after pollination obtained by Liu et al. (Liu et al., 2016). The genotype data consisted of 1.25 M SNPs (B73_RefGen_v2) covering the whole maize genome with a minimum allele frequency (MAF) ≥ 0.05 (Liu et al., 2016). Here, the GLM approach controlling population structure (Q) was adopted after comparing the performances of three linear models, that is, GLM (GLM + Q, only control population structure), MLM (GLM + K, only control relative kinship) and MLM (GLM + Q + K, correcting for population structure and relative kinship) models (Li et al., 2013) implemented in TASSEL 3.0 (Bradbury et al., 2007) was therefore used to perform GWAS. For GLM model, y = Xα + Zβ + e. or y = SNP + Q + e. For MLM model, y = Xα+ Zβ + Wμ+ e. or y = SNP + Q + Kinship + e. where, y is the trait value, Xα (population structure or Q matrix) is fixed effect, Zβ (SNP or marker effect) is fixed effect, Wμ (Kinship matrix) is random effect and e is residual (Yu et al., 2006). In addition, to control the type I (false positive) and type II (false negative) error rates, Quantile-Quantile (QQ) plots of the three statistical models for each LA-related trait were compared, if a model that has a distribution closer to the diagonal line indicates a better control for type I and II errors (Zhang et al., 2010). Thus, the more appropriate model was selected to interpret the GWAS results of LA-related traits.

Taking into account the linkage disequilibrium (LD) among SNP markers, the effective marker number (En) for the genotypic dataset was 490 548, as previously calculated using GEC software (Deng et al., 2017). The suggested *p*-value of 2.04 × 10^–6^ (1/En) was used as the genome-wide threshold for significant SNP–trait associations, as commonly used in plant genome-wide association studies.

### Meta-analyses

Based on a published review (Cao et al., 2022), the QTL information have been reported for LA-related traits (Lu et al., 2007; Ku et al., 2010; Tian et al., 2011; Ku et al., 2012; Chen et al., 2015; Ding et al., 2015; Hu et al., 2015; Li et al., 2015; Yang et al., 2015; Ku et al., 2016; Pan et al., 2017; Shi et al., 2017; Wang et al., 2017; Dzievit et al., 2019; Liu et al., 2019; Zhang et al., 2019; Zhang et al., 2020; Tang et al., 2021). The QTL information (Chromosome, LOD, R^2^, Confidence Interval and so on) were summarized from eighteen studies published in the lasted 15 years. For QTLs whose confidence interval (CI) was unknown, the following formulas were used to calculate CI:

$$CI=530/\left(N\times R^{2}\right)$$ (1)

$$CI=163/\left(N\times R^{2}\right)$$ (2)

where CI is the confidence interval, *N* is the number of materials in the mapping population, and *R* ^2^ is the phenotypic variation. Eq. 1 was used for Backcross and F_2_ mapping populations, and Eq. 2 was used for recombinant inbred line (RIL) mapping populations (Darvasi and Soller, 1997). If the LOD value was unknown, it was calculated using *R* ^2^ = 1–10^(−2LOD/*N*)^ (Nagelkerke, 1991; Liu, 1997). QTLs without *R* ^2^ information were discarded.

IBM2 2008 Neighbors (MaizeGDB, https://www.maizegdb.org/) (Sharopova et al., 2002) was used as a reference genetic map, and 19 051 high-density markers (SSR, RFLP, RAPD, and SNP) covering the whole maize genome were obtained. The markers were combined with the collected QTLs and genetic map information, and a meta-analysis was performed to analyze all QTLs and markers in the genome and obtain the most suitable number of QTLs (Veyrieras et al., 2007). Five different models (1-, 2-, 3-, 4-, or N-QTL) with different Akaike information criterion (AIC) values were proposed and used in BioMercator V4.2.3, a genetic map compilation and meta-analysis software to integrate QTL data with genome structural and functional annotation. The model with the lowest AIC-value was considered optimal (Arcade et al., 2004). Finally, the QTLs presented by the optimum model were regarded as the meta-QTLs, we named these meta-QTLs using the format M-q-trait-chromosome number-sequence number [for example, *MqLA1-1*, *MqLA* (Leaf Angle) *1* (chromosome number) *-1* (sequence number)]*.*

### Analyses of candidate genes

The previously estimated decay distance of LD in this AMP (∼30 kb, *R* ^2^ = 0.1) (Liu et al., 2016) was used to define a 60-kb QTL interval, i.e., the 30 kb upstream and downstream of each SNP. In each environment and for each trait, all QTLs with overlapping QTL intervals were categorized as non-redundant QTLs. If a non-redundant QTL was detected by different LA-related traits, in different environments, from previously published LA QTLs, or physical distance of less than 10 Mb by GWAS and meta-QTL analysis, it will be defined as a co-located QTL. All potential candidate genes within all non-redundant QTLs or co-located QTLs were identified based on the filtered working gene list from the reference genome of the maize inbred line B73 (RefGen_v2). These genes were downloaded from MaizeGDB and annotated using InterProScan (http://www.ebi.ac.uk/interpro/scan.html). Phenotypic variation explained (PVE) by each QTL was estimated based on the R^2^ value of the most significant SNPs within the QTL. Candidate genes within the CIs of meta-QTLs were also obtained. In each non-redundant QTL or co-located QTL, the most likely candidate gene was selected based on its annotation or because it contained the peak SNP (the most significant SNP). If there was no gene in the interval, the neighboring gene of the peak SNP was considered to be the most likely candidate gene.

### Gene Ontology enrichment analyses

Gene Ontology (GO) enrichment analysis was performed using OmicShare tools (https://www.omicshare.com/tools) (Ding et al., 2019). Specifically, the candidate genes were mapped to the various sets of the GO database (http://www.geneontology.org/), the number of genes in each set was counted, and the list of genes with a specific GO function and the number of genes were obtained. The top 20 GO terms with minimum *p* values were selected for analysis and plotting (Lv et al., 2019).

### Linkage disequilibrium analyses

Linkage disequilibrium (LD) was estimated by the squared correlation of the paired SNPs, which was calculated with TASSEL 3.0 software. An LD plot was generated using the ‘genetics’ and ‘LDheatmap’ package in R (version 4.1.1).

### Haplotype analyses

All SNPs (MAF ≥0.05) in target genes (*ZmULA1*, *ZmCLA4*, *lg1*, *lg2* and *ZmTAC1*) were used for haplotype analysis by using 1.25M SNPs genotype data (Liu et al., 2016). The BLUP values of LA-related traits in 492 inbred lines were used as phenotypic data. Haplotypes contained in more than 10 inbred lines were used for comparative analysis.

## Results

### Phenotypic evaluation

LA at three positions, ULA, MLA and LLA (Supplementary Figure S1), were investigated in five environments. The LA-related traits showed the greatest variation in YY and lowest variation in YC. The maximum angle was 8.7-fold higher than the minimum angle (9.90°–86.50°) for ULA, 4.5-fold higher for MLA (14.05°–63.34°), and 3.8-fold higher for LLA (19.48°–74.00°) (Table 1). Thus, the panel exhibited rich genetic diversity in LA, which could be ranked LLA > MLA > ULA (Table 1 and Supplementary Figure S2). All phenotypes were typical quantitative traits, in that they were continuous variables that exhibited a normal distribution (Supplementary Figure S3).

**TABLE 1:** Descriptive statistics for leaf angle-related traits of maize in the association mapping panel in different environments.

Trait	Environment	Range (°)	Mean (°)	sd.	Ske.	Kur.	*H* ^*2*^
ULA	HB	11.65–67.56	34.65	11.57	0.61	0.08	0.59
	YC	14.32–62.98	37.65	10.47	0.17	−0.56	
	YY	9.90–86.50	31.93	14.19	1.17	1.60	
	YYC	11.48–72.65	33.11	11.49	0.69	0.59	
	YYX	6.71–80.08	35.11	12.56	0.74	0.60	
	BLUP	14.71–61.08	35.08	8.37	0.58	0.21	
MLA	HB	16.95–63.60	38.08	9.31	0.43	−0.03	0.71
	YC	14.05–63.34	38.26	9.97	0.18	−0.45	
	YY	18.68–79.68	39.51	11.67	0.85	0.74	
	BLUP	23.31–60.31	38.77	6.72	0.58	0.36	
LLA	HB	23.42–77.64	43.43	10.39	0.72	0.68	0.69
	YC	19.62–68.87	42.00	9.21	0.14	−0.13	
	YY	19.48–74.00	43.87	9.95	0.37	−0.04	
	BLUP	30.81–62.95	43.19	5.69	0.46	0.49	

sd., STDEVP, standard deviation calculated based on the given sample population.

Ske., SKEW, the degree of asymmetry used to represent the relative mean.

Kur., KURT, a peak value used to represent the dataset.

*H* ^*2*^, Broad-sense heritability.

**FIGURE 1:**
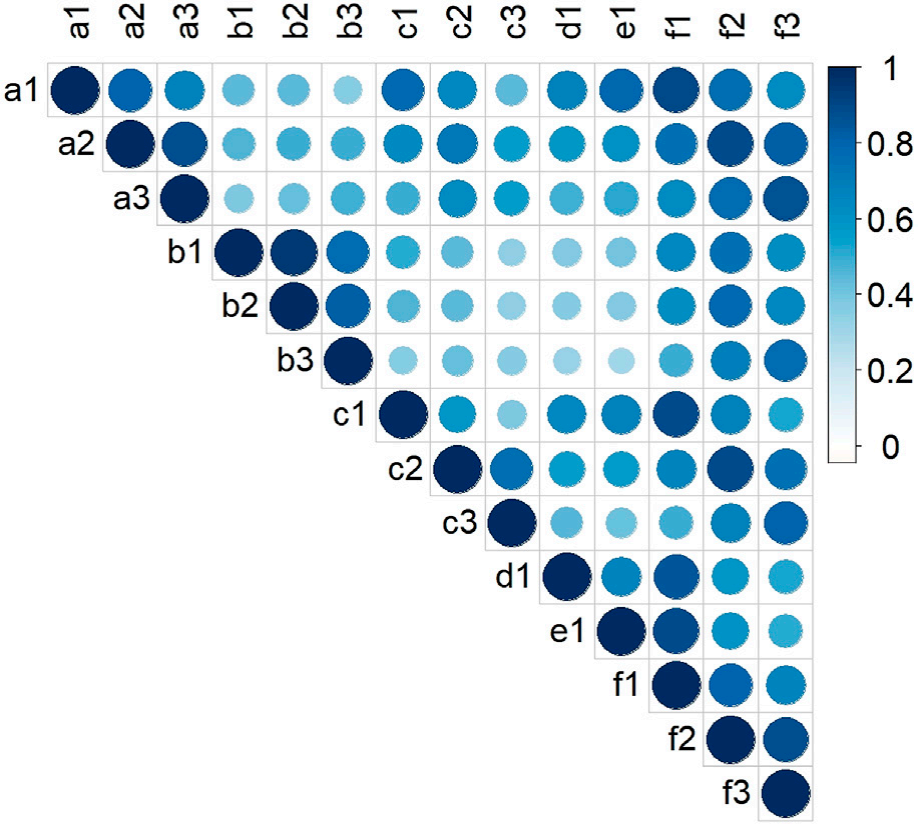
Pearson correlation coefficients for LA-related traits of maize in the association mapping panel in different environments. **(a‒f)**: HB, YC, YY, YYC, YYX, and BLUP, respectively; (1–3): ULA, MLA, and LLA, respectively.

Two-way ANOVA indicated that there were significant genetic (G) and environmental (E) effects on LA-related traits, but the effect of their interaction (G × E) was not significant (Supplementary Table S1). The results also showed that genetics had a greater effect on LA than environment. There were positive correlations between LA-related traits in all environments (Figure 1). The broad-sense heritabilities of ULA, MLA, and LLA were 0.59, 0.71, and 0.69, respectively (Table 1), again indicating that they were mainly affected by genetic factors.

### GWAS

LA was less sensitive to the K model than other two models, and this model could better control type I and type II errors (Supplementary Figure S4). In addition, we examined the distribution of the three LA traits in each sub-population across different locations, the results shown that population structure has a small effect on the leaf angle (Supplementary Figure S5). Therefore, the GWAS under the K model was analyzed further (Supplementary Figure S6).

**FIGURE 2:**
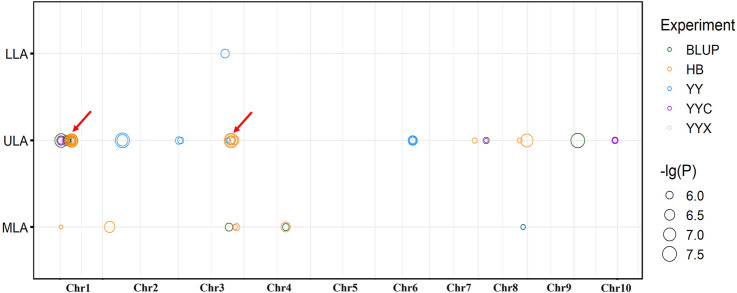
Distribution of significant loci detected by GWAS on maize chromosomes. Red arrows indicate co-located QTLs, for the same trait in different environments.

In total, 85 significant SNPs were detected by ULA, MLA and LLA in all environments, and involving 32 non-redundant QTLs. The QTLs were distributed on all chromosomes except chromosome 5 and 9, and there was a QTL hot spot at chromosome 1 and 3 (Figure 2). Twenty-one major-effect QTLs explained more than 10% of the phenotypic variation (*R* ^2^ = 10.01–24.09%). Twenty-six QTLs were identified for ULA, with a mean *R* ^2^ of 9.34% (4.80%–15.16%), and seven QTLs were detected for MLA, with a mean *R* ^2^ of 15.14% (9.48%–24.09%). Only one QTL was associated with LLA, and it explained 11.63% of the phenotypic variation.

Fourteen non-redundant QTLs were detected in HB and explained 9.08%–21.93% of the phenotypic variation. Ten non-redundant QTLs were detected in YY (10.05%–15.16%), nine in BLUP (4.80%–24.09%), and only four and two in YYC and YYX, respectively. No significant QTLs were detected in YC (Figure 2).

Five QTLs were identified in multiple environments (Figure 2). For ULA, *qLA2* was detected in YYC, YYX, and BLUP, and *qLA3* and *qLA27* were detected in YYC and BLUP. For MLA, *qLA23* was identified in HB and BLUP, and *qLA29* was detected in BLUP and YY. These loci can be stably inherited in different environments and play an important role in the regulation of plant architecture. QTLs detected for different traits may have pleiotropic effects. *qLA1* was identified by MLA and ULA and may therefore regulate the size of both ULA and MLA. In addition, *qLA21* was also detected in different traits (ULA and MLA), suggesting that *qLA21* may have a similar role in the regulation of LA at different positions. Detailed information on the GWAS results, including the physical position, *p*-value, and *R* ^2^ for each QTL is provided in Supplementary Table S3.

### Candidate gene analysis

Based on the B73 RefGen_v2 reference genome (https://www.maizegdb.org/), 82 genes were found within 32 non-redundant QTLs. For example, *GRMZM2G049159* within *qLA7* was identified for ULA in HB and encodes a GRAS family transcription factor; its homolog *OsGRAS23* induces downstream stress-responsive genes and positively regulates drought tolerance in rice (Xu et al., 2015), and it may be involved in the regulation of rosette leaf development in *Arabidopsis* (Schmid et al., 2005). *qLA18* was also identified for ULA in HB, and four genes are located in this QTL. Among them, *GRMZM2G071705* encodes an F-box protein; this type of protein may be involved in the strigolactone (SL) biosynthetic pathway and further regulate plant architectural traits such as tiller number and LA (Ishikawa et al., 2005; Dong et al., 2016).

Twenty genes were identified in co-located QTLs and may be involved in protein ubiquitination (*GRMZM2G134176*), carbon metabolism (*GRMZM2G134256*), and the citric acid cycle (*GRMZM2G158378*) and play important roles in the regulation of plant growth and stress tolerance. *GRMZM2G311328* is located in *qLA21*, which was identified for MLA and ULA in HB. This gene may be pleiotropic; its homolog encodes a vesicle auxin mediated transporter, and regulation of auxin polar transport is important for development and architecture of *Arabidopsis* (Kitakura et al., 2011). *GRMZM2G134073* in *qLA29* was simultaneously identified for MLA in BLUP and YY; it encodes a NAC family transcription factor that can promote resistance to heat damage (Li H et al., 2020). Its homolog *OsNAC10* plays a key role in rice drought and disease resistance (Jeong et al., 2010), and it may also participate in regulating plant architecture and abiotic stress tolerance.

Among the 82 genes, only *GRMZM2G071790* has been reported, it encodes a beta-6 tubulin and plays an important role in maize tolerance to *Ustilago maydis* (Ruan et al., 2021), as well as catalyzing auxin transport in *Arabidopsis* (Terasaka et al., 2005). Although most of these genes screened by GWAS have unknown functions, their homologous genes can provide valuable information. Interestingly, some genes may have pleiotropic effects, that may play important roles at both LA and stress resistance. Thus, our results provide new and valuable information for understanding the genetic mechanisms that underlie LA.

### Meta analysis

**FIGURE 3:**
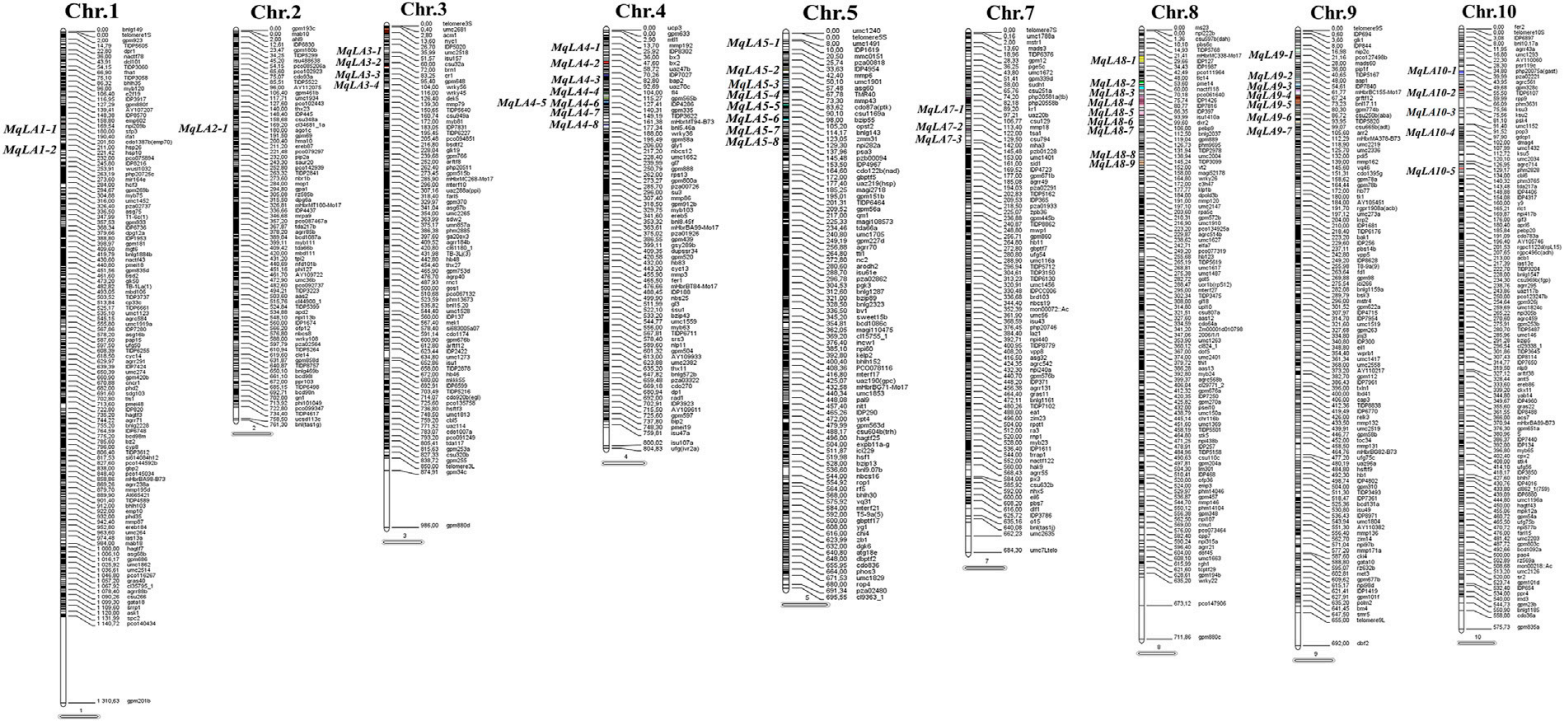
Meta-QTLs distributed on maize chromosomes with IBM2 2008 Neighbors as a reference.

**FIGURE 4:**
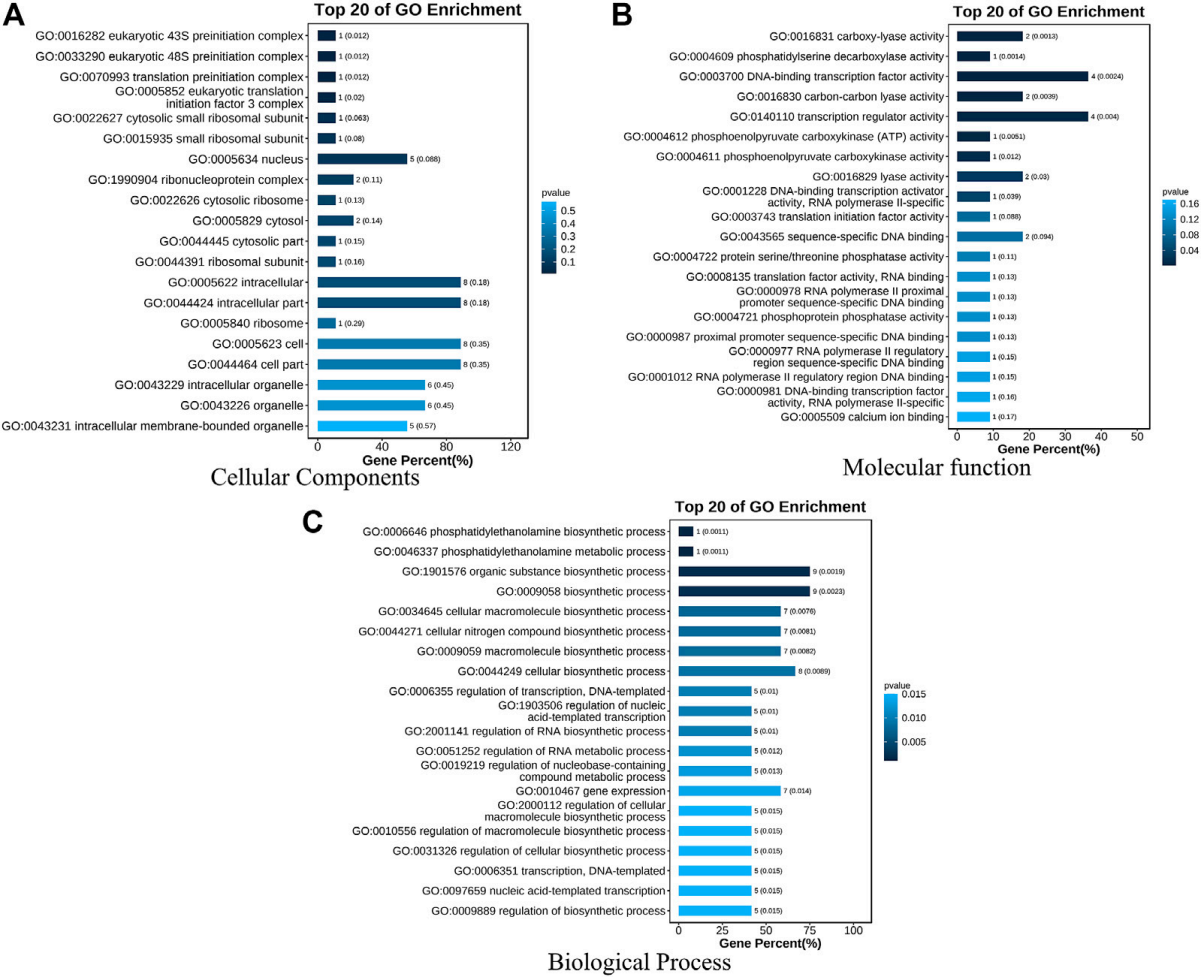
GO enrichment analysis of seventeen genes within seven co-localized QTLs. Three GO Ontologies are shown **(A)** Cellular Component; **(B)** Molecular Function, and **(C)** Biological Process.

A total of 294 QTLs related to LA were obtained from studies published in the last 15 years (Supplementary Table S4) (Cao et al., 2022). 47 meta-QTLs were obtained by the meta-QTL analysis, containing 816 genes. These 47 QTLs were distributed on all chromosomes except chromosome 6 (Table 2 and Figure 3). The largest number of meta-QTLs (9) were located on chromosome 8, and only one was located on chromosome 2. Seven co-located QTLs were identified by both GWAS and meta-analysis (Table 3). Based on the GWAS results, 17 genes within the seven co-located QTLs were examined, but none had been characterized previously. Gene Ontology enrichment was then performed with the 17 genes, and they were mainly involved in the synthesis of intracellular parts (cellular component, GO: 0044424), transcriptional regulatory activity (molecular function, GO:0140110) and organic substance biosynthetic process (biological process, GO:1901576) (Figure 4). Interestingly, LA-related genes identified to date encode transcription factors or participate in hormonal signal transduction (Cao et al., 2022), and plant hormones are natural organic compounds (Adam 1999). These results suggest that the 17 genes in the co-localized QTLs may regulate LA by encoding transcription factors and/or participating in the synthesis of organic compounds, including plant hormones.

**TABLE 2:** List of meta-QTLs and accompanying details for leaf angle-related traits in maize.

Meta QTL	Chr	Position (cM)	CI (cM)	CI(Mb)	Left marker	Right marker
*MqLA1-1*	1	181.08	180.53–181.63	24.58–25.28	sfp3	cdo860a
*MqLA1-2*	1	209.46	209.18–209.75	32.87–33.12	bnlg176	xyl5
*MqLA2-1*	2	169.93	169.92–169.94	15.57–16.04	msl2	hon101
*MqLA3-1*	3	50.37	50.27–50.47	3.56–3.82	cdo511	isu157
*MqLA3-2*	3	63.28	62.42–64.15	4.19–4.36	IDP8355	cl19880_1
*MqLA3-3*	3	92.58	90.70–94.46	6.94–7.41	T3-9 (8447) (3)	eif3
*MqLA3-4*	3	107.90	107.66–108.14	8.59–8.89	csu728c	gts1 (CBM 3.03)
*MqLA4-1*	4	12.20	4.80–19.60	1.09–1.99	bnlg1434	IDP4473
*MqLA4-2*	4	61.87	59.77–63.97	3.75–4.09	uaz52a	pd1
*MqLA4-3*	4	88.11	85.62–90.60	5.25–5.38	TIDP2802	uaz61a
*MqLA4-4*	4	106.32	105.69–106.95	5.54–9.80	uaz184 (hfi)	umc1288
*MqLA4-5*	4	108.76	108.07–109.45	6.44–7.57	cle7	mads25
*MqLA4-6*	4	122.55	120.36–124.75	10.02–10.90	nbcs11	IDP4286
*MqLA4-7*	4	133.29	131.80–134.79	10.46–11.92	gpm574a	bnlg1126
*MqLA4-8*	4	183.50	181.95–185.05	17.50–17.86	umc1902	wrky36
*MqLA5-1*	5	10.10	4.20–16.00	0.54–0.88	telomere5S	AY109758
*MqLA5-2*	5	46.73	46.29–47.17	2.14–2.59	IDP7849	umc1901
*MqLA5-3*	5	53.24	52.52–53.96	2.32–2.59	IDP2557	prh24
*MqLA5-4*	5	61.67	61.12–62.23	2.70–2.86	umc2591	phm5359
*MqLA5-5*	5	76.90	75.53–78.28	3.73–4.22	bhlh159	IDP1463
*MqLA5-6*	5	91.07	89.73–92.41	4.88–5.49	IDP6013	TIDP5654
*MqLA5-7*	5	108.57	107.26–109.59	6.14–6.67	uaz163	gpm921a
*MqLA5-8*	5	132.51	132.26–132.76	7.13–7.36	ucsd64a	npi305a
*MqLA7-1*	7	91.36	90.65–92.08	6.60–7.45	ao5	bnlg2160a
*MqLA7-2*	7	116.55	115.62–117.49	10.52–10.78	gpm913a	magi108570
*MqLA7-3*	7	130.22	129.59–130.86	13.96–14.08	csu794	y8
*MqLA8-1*	8	34.72	30.97–38.47	3.66–4.24	TIDP3564	arf4
*MqLA8-2*	8	61.95	60.99–62.91	7.04–7.30	nactf118	nactf130
*MqLA8-3*	8	68.71	67.00–70.42	5.68–6.70	gpm600	TIDP5156
*MqLA8-4*	8	74.72	72.37–77.07	6.03–6.86	TIDP5156	IDP1629
*MqLA8-5*	8	79.01	78.34–79.69	6.86–7.98	ncr (sod3b)	IDP1629
*MqLA8-6*	8	92.51	91.91–93.11	7.52–8.09	TIDP3314	isu1410a
*MqLA8-7*	8	98.12	95.78–100.47	8.11–8.89	gpm932d	IDP7980
*MqLA8-8*	8	156.01	153.42–158.61	17.24–18.37	cle26	cdo328
*MqLA8-9*	8	164.53	164.51–164.56	18.20–19.85	wrky80	wrky26
*MqLA9-1*	9	23.06	20.41–25.72	5.01–5.52	rz144c	mads60
*MqLA9-2*	9	53.11	52.40–53.83	8.23–8.94	bzip100	TIDP4624
*MqLA9-3*	9	61.86	59.63–64.09	8.36–9.74	mHbrMG162-Mo17	c1
*MqLA9-4*	9	68.73	67.03–70.43	9.75–10.85	umc113a	crs4a
*MqLA9-5*	9	78.53	73.55–83.51	10.85–11.57	isu1146	ptf1
*MqLA9-6*	9	93.27	91.91–94.63	11.93–12.35	mkk1	TIDP5270
*MqLA9-7*	9	103.18	102.71–103.65	12.98–13.45	umc1131	chr113
*MqLA10-1*	10	41.83	40.53–43.13	2.78–3.06	pza02221	agrc561
*MqLA10-2*	10	58.78	57.86–59.71	4.07–4.17	cl24029_1	rpp9
*MqLA10-3*	10	101.90	95.60–108.20	5.38–5.92	glk5	TIDP3571
*MqLA10-4*	10	133.65	132.93–134.38	7.40–9.23	umc2749	oy1
*MqLA10-5*	10	187.8	187.45–188.15	58.30–59.51	gpt1	mHbrMC413-Mo17

**TABLE 3:** List of co-located QTLs and physical position revealed by GWAS and meta-analysis.

Chr	GWAS		Meta-analysis	
	QTL name	CI (Mb)	QTL name	CI (Mb)
1	*qLA3*	23.53–23.59	*MqLA1-1*	24.58–25.28
1	*qLA6*	35.71–35.77	*MqLA1-2*	32.87–33.13
1	*qLA7*	40.11–40.17	*MqLA1-2*	32.87–33.13
1	*qLA8*	40.15–40.21	*MqLA1-2*	32.87–33.13
3	*qLA12*	1.95–2.01	*MqLA3-1*	3.56–3.82
3	*qLA13*	8.06–8.12	*MqLA3-4*	8.59–8.89
8	*qLA27*	28.27–28.33	*MqLA8-9*	19.83–19.85

**FIGURE 5:**
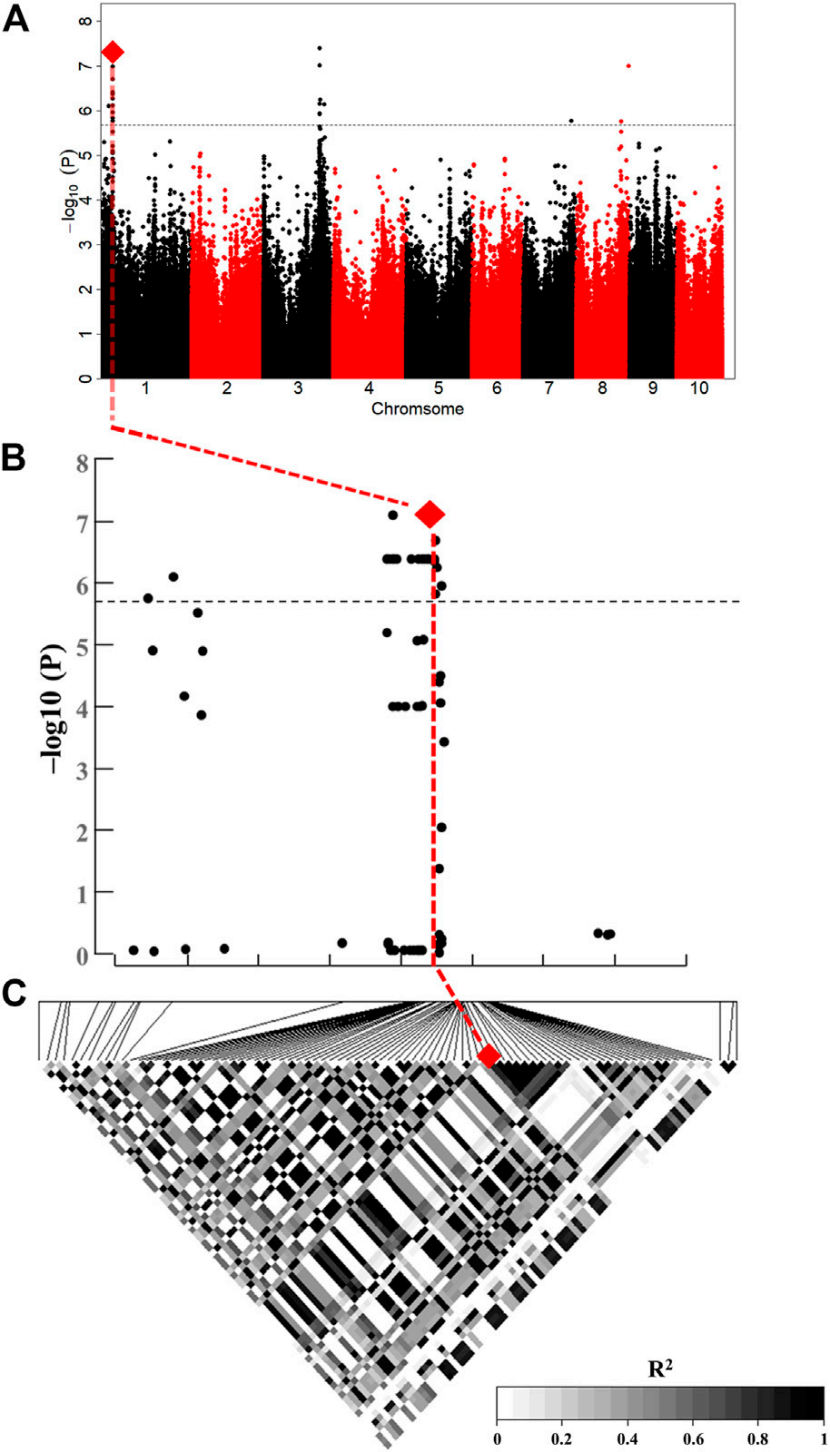
*ZmULA1* affected LA-related traits. **(A)** Manhattan plot of ULA at HB. The line represents the threshold −log_10_(P) ≥ 5.69 (*p* ≤ 2.04 × 10^–6^). **(B)** Enlarged Manhattan plot of the lead SNP and 85 SNPs associated with *ZmULA1*; red diamonds represent the lead SNP. **(C)** *R* ^2^ values of significant SNPs associated with *ZmULA1*; the lead SNP was located in 2.64 kb downstream of *ZmULA1*.

*qLA7*, which was related to ULA, was located on chromosome 1 and had an extremely strong association signal (Figure 5A and Figure 5B). There was only one gene (*GRMZM2G049159*) within this QTL, and it encodes a GRAS family transcription factor, involved in the synthesis of organic (GO:1901576) (Figure 4), plant hormones such as CK and BR, as natural organic compounds, play an important role in regulating LA. Therefore, *ZmULA1* may be involved in the metabolic process of plant hormones to regulate maize LA. Additionally, its homolog may be involved in the development of rosette leaves in *Arabidopsis* (Schmid et al., 2005). We speculate that it may affect the formation of LA in maize, and we named it *ZmULA1*.

### Haplotype analysis of *ZmULA1*

**FIGURE 6:**
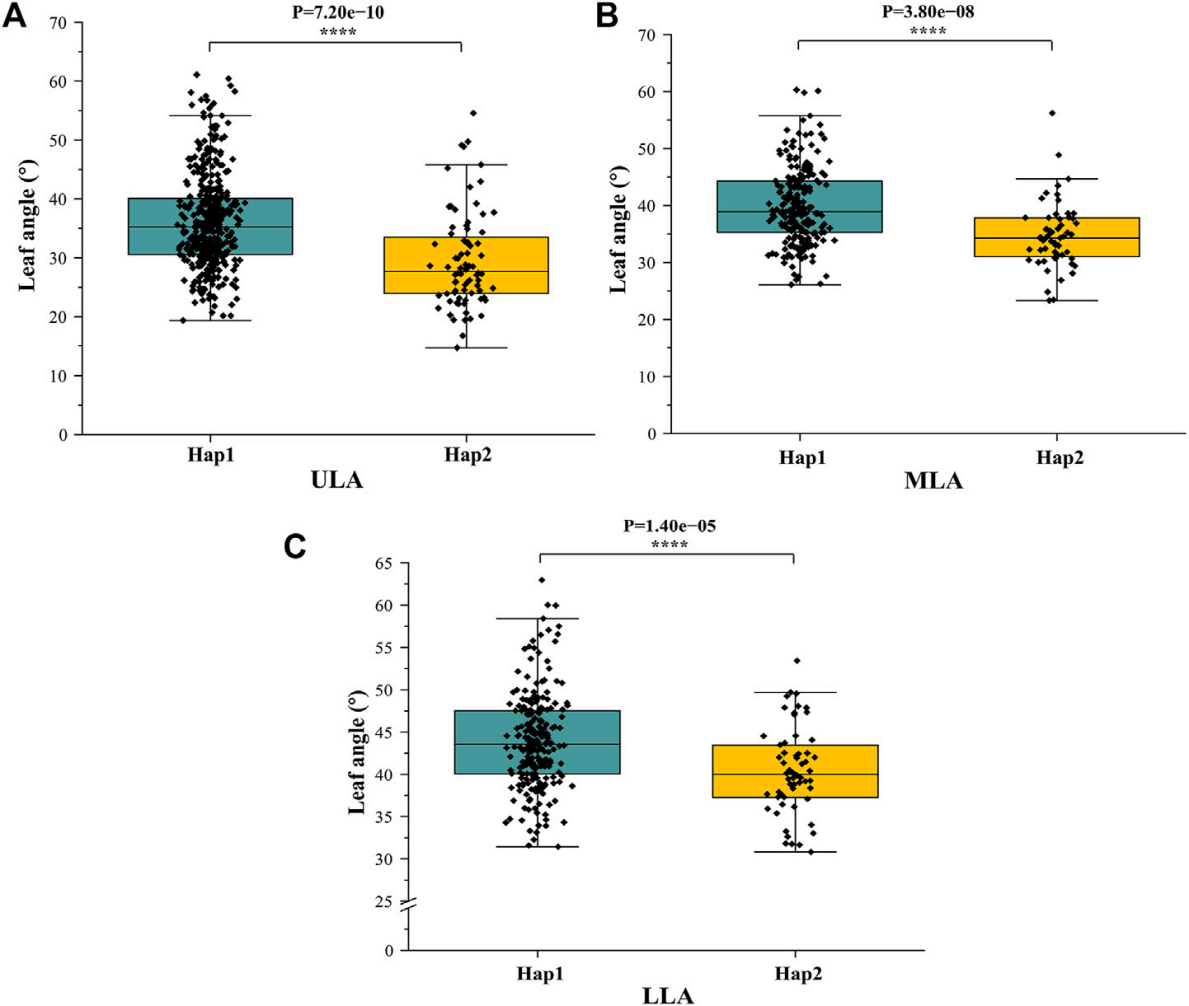
Differences in LA-related traits between Hap1 and Hap2. **(A)** ULA. **(B)** MLA. **(C)** (LLA). *: *p* < 0.05, **: *p* < 0.01, ***: *p* < 0.001, ****: *p* < 0.0001.

**FIGURE 7:**
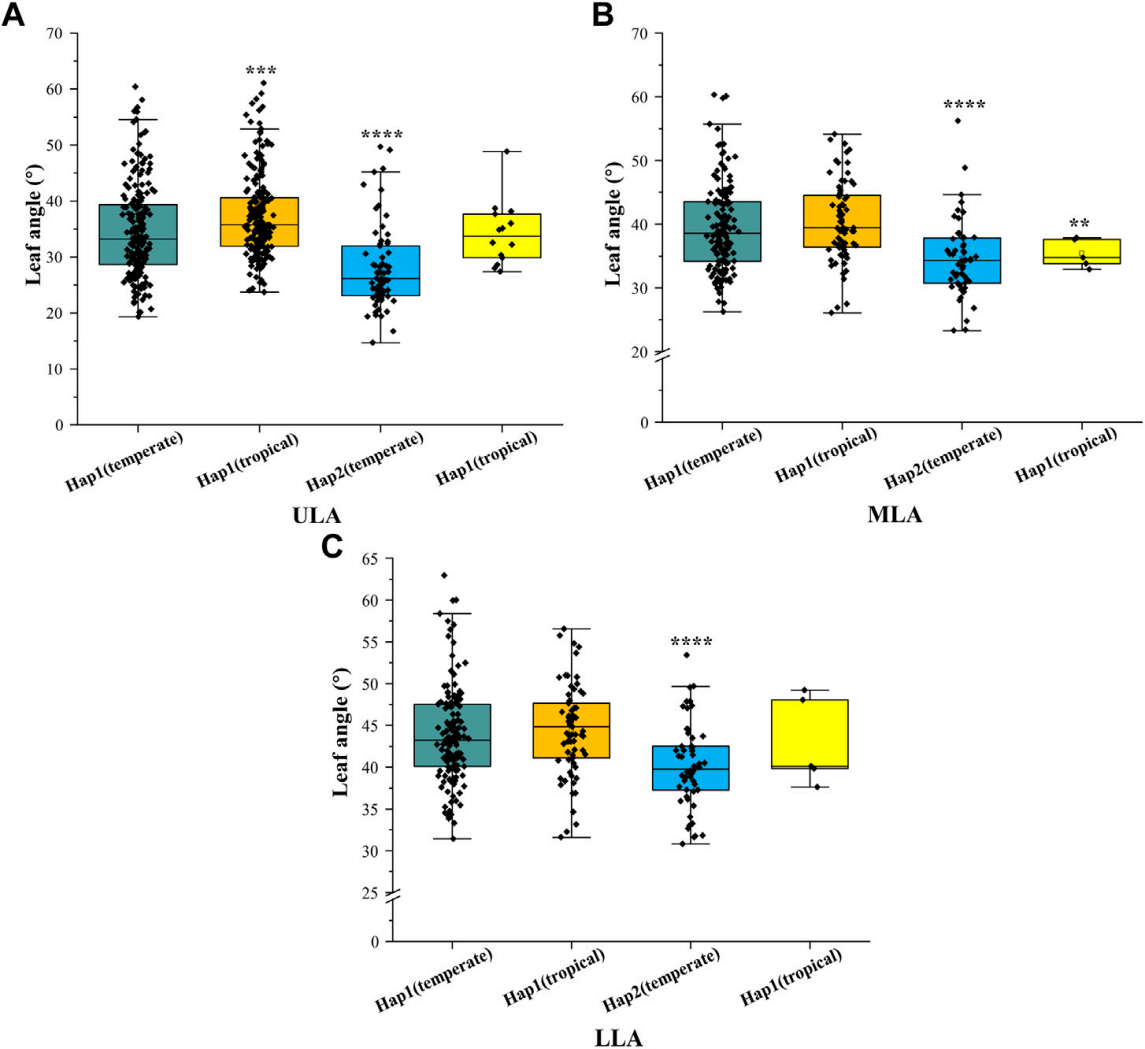
Differences in LA-related traits between Hap1 (temperate), Hap1 (tropical), Hap2 (temperate), and Hap2 (tropical). **(A)** ULA. **(B)** MLA. **(C)** (LLA). The significance level was obtained by comparing others haplotypes with Hap1(temperate), respectively. *: *p* < 0.05, **: *p* < 0.01, ***: *p* < 0.001, ****: *p* < 0.0001, not marked stands for not significant.

**FIGURE 8:**
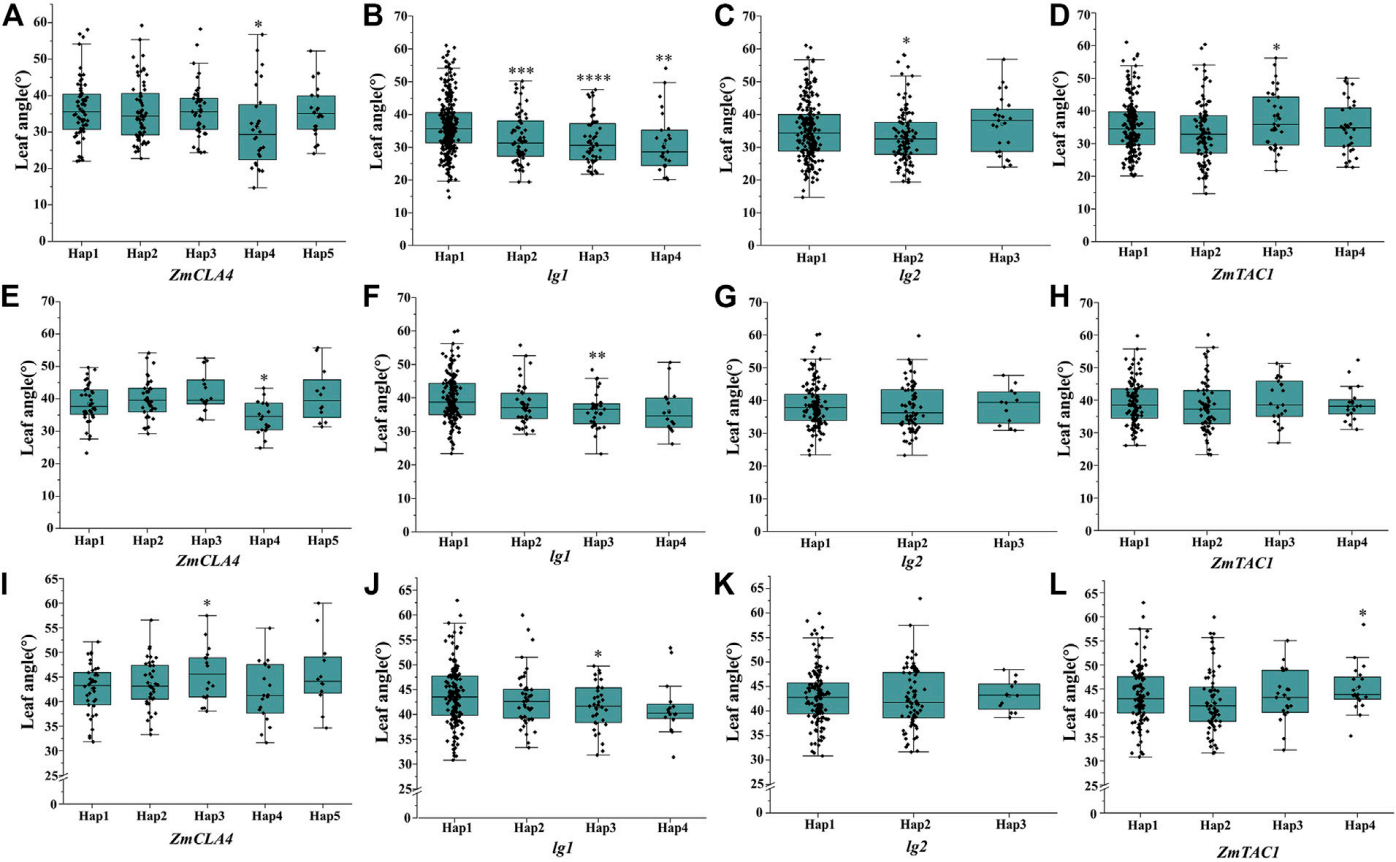
Differences of haplotypes in LA-related traits within four cloned genes that regulate maize LA. **(A–D)**, ULA, of *ZmCLA4*, *lg1*, *lg2*, *ZmTAC1*, respectively; **(E–H)**, MLA, of *ZmCLA4*, *lg1*, *lg2*, *ZmTAC1*, respectively; **(I–L)**, LLA, of *ZmCLA4*, *lg1*, *lg2*, *ZmTAC1*, respectively. The significance level was obtained by comparing others haplotype with hap1, respectively. *: *p* < 0.05, **: *p* < 0.01, ***: *p* < 0.001, ****: *p* < 0.0001, not marked stands for not significant.

To analyze the haplotype of *ZmULA1*, we extracted all polymorphic sites within one LD decay distance near the lead SNP (chr1.S_40141659, *p* = 7.57e−08) to perform LD analysis, there was a strong linkage relationship (the average pairwise *r* ^2^ value was 0.45) between the lead SNP and polymorphic sites (Figure 5C). Subsequent analysis of 17 SNPs in *ZmULA1* using BLUP values of 492 lines identified two haplotypes. All maize inbred lines belonged to hap1 (412) or hap2 (80), and the mean value of hap2 was smaller than that of hap1 for the three LA-related traits (Supplementary Table S5). Specifically, ULA (*p* = 7.20e−10), MLA (*p* = 3.80e−08), and LLA (*p* = 1.40e−05) showed extremely significant differences between hap1 and hap2 (Figure 6). Temperate materials had smaller values of LA-related traits (ULA, MLA and LLA) than tropical lines, and the temperate materials with hap2 had the smallest LAs (Figure 7). Furthermore, 65% (52/80) of the maize inbred lines derived from China belonged to hap2 (Table 4), and these elite inbred lines can be used to improve the plant architecture of maize cultivars. In summary, these results suggest that natural variation in *ZmULA1* may affect the three LA-related traits, which may be influenced by constant selection during maize breeding. In addition, we conducted haplotype analysis of four known genes (*ZmCLA4*, *lg1*, *lg2* and *ZmTAC1*) that regulate LA in previous studies (Harper and Freeling, 1996; Moreno et al., 1997; Yu et al., 2007; Zhang et al., 2014), favorable haplotype for each gene which have smallest LA was identified, that are hap4 for *ZmCLA4*, hap4 for *lg1*, hap2 for *lg2* and hap2 for *ZmTAC1* (Figure 8). The germplasms with favorable haplotype combinations of 492 inbred lines were identified, and a trend was found that the more favorable haplotypes, the smaller the leaf angle (Supplementary Table S6).

**TABLE 4:** The number of haplotypes for *ZmULA1* in 492 maize inbred lines classified by origin and source, respectively.

Haplotype	Total	Origin			Source		
		Temperate	Tropical/sub-tropical	China	United States	CIMMYT	Other
Hap1	412	202	210	166	46	195	5
Hap2	80	65	15	52	14	14	0

## Discussion

Increasing planting density is an important way to improve maize yield, and LA is one of the key traits that determine whether maize can tolerate high planting density. Several genes that regulate maize LA have been cloned in previous studies. For example, *lg1* (liguleless1) encodes a protein containing an SBP domain, and its mutation causes the loss of tongues and ears, making plants more compact (Moreno et al., 1997). *lg2* (liguleless2) encodes a basic leucine zipper (bZIP) transcription factor. These two genes are located in the same developmental pathway, in which *lg2* plays an earlier role than *lg1* (Harper and Freeling, 1996; Walsh et al., 1998). *ZmTAC1* is also considered to regulate LA in maize. It encodes a protein composed of 263 amino acids that is most highly expressed in the maize leaf sheath, and the difference in LA traits between the compact leaf inbred line Yu82 and the extended leaf inbred line Shen137 is caused by nucleotide variation in its non-coding region (Ku et al., 2011). Recently, Li et al. cloned *ZmACS7* from the *Sdw3* mutant; its overexpression caused lower plant height and higher LA, and multi-omics analysis showed that it alters plant architecture by promoting growth of the auricle and inhibiting elongation of the internode cells (Li Z et al., 2020). The genetic and molecular mechanisms of LA have also been widely dissected in other crops. For example, *OsTAC1* in rice is a homolog of maize *ZmTAC1* and has an intron sequence AGGA in the 3′ non-coding region. A single SNP variation (A/G) that changes ‘AGGA’ to ‘GGGA’ can lead to reduced expression of *ZmTAC1* and an LA close to zero, making the rice plant more compact (Yu et al., 2007).

In this study, 85 significant SNPs in 32 QTLs were identified by GWAS and included 82 genes. Seven QTLs could be co-located in different environments or different LA-related traits (Figure 2), they are considered as genomic hot regions and worth to mining for valuable information. *qLA1* and *qLA21* were co-located by ULA and MLA and they explained 16.96% and 9.48% of phenotypic variation, respectively. Only one gene (*GRMZM2G158378*) was found within *qLA1*, and its rice homolog has been shown to promote the transport and absorption of silicon (Mitani et al., 2009). Five genes were located in *qLA21*, one of which encoded a glycosyltransferase (*GRMZM2G014770*) and another a vesicle-mediated substance transporter (*GRMZM2G311328*). Five co-located QTLs (*qLA2*, *qLA3*, *qLA23*, *qLA27*, and *qLA29*), explaining 6.13%–18.89% of phenotypic variation, were identified in at least two environments, indicating that they are genetically stable and less affected by the environment. Most QTLs (∼84%) were detected in only one environment (27/32); a possible explanation is that LA is a complex quantitative trait regulated by a large number of minor genes that are greatly affected by the environment, leading QTL effects to vary in different environments. It is worth mentioning that 21 of the 32 QTLs (∼66%) were major QTLs with *R* ^2^ values greater than 10%. These results provide new information for understanding the genetic basis of the natural variation in LA.

In the current study, we collected QTLs for LA published over the past 15 years. In total, 294 QTLs were obtained and mapped to the IBM2 2008 Neighbors genetic map; 47 meta-QTLs were then identified by meta-analysis (Figure 3 and Table 2). Meta-analysis can effectively narrow the confidence interval of QTL and improve the prediction accuracy of candidate genes (Goffinet and Gerber, 2000; Arcade et al., 2004). Moreover, 816 genes were examined in 47 meta-QTLs; fifteen had been reported previously, and five had been functionally characterized. Two genes (*GRMZM2G005066*, *c1* and *GRMZM2G089713*, *sh1*) were involved in kernel colored aleurone and endosperm development (Tohge et al., 2017; Zhou et al., 2021). *GRMZM2G141399* (*du1*) encoded starch synthase III, which makes kernels glassy and inhibits glycogen accumulation (Cao et al., 1999). Two additional genes (*GRMZM2G051637*, *cr4* and *GRMZM2G419806*, *oy1*) were associated with plant height and senescence of maize seedlings (Peiffer et al., 2014; Khangura et al., 2020). Notably, *AC195340.3_FG001* (*tua1*), located in *MqLA5-6*, is associated with plant architecture at different planting densities and encodes an alpha tubulin family protein; it has been annotated but not functionally characterized (Incognito et al., 2020). These results suggested that meta-analysis can provide more valuable information.

Seven QTLs were jointly identified by GWAS and meta-analysis (Table 3), and two, *qLA3* and *qLA7*, had significant association signals at chromosome one by GWAS (Figure 5A). Three genes (*AC205725.3_FG010*, *GRMZM2G471253*, and *GRMZM2G171073*) were located in *qLA3* and were expressed in leaves, potentially affecting leaf development. *GRMZM2G171073* encodes a C2H2-like zinc finger protein, and its homolog IDD1 is involved in gibberellin (GA) signal transduction and transport in *Arabidopsis* (Fukazawa et al., 2014). GA affects plant structure and interacts with BR, causing variation in LA (Tang et al., 2018). *ZmULA1* was located in *qLA7*, and hap1 and hap2 for this gene showed highly significant differences in LA-related traits (Figure 6). Hap2 was considered as a favorable haplotype because the hap2 had smaller LA (ULA, MLA and LLA) than other haplotypes, and temperate germplasms with hap2 had the smallest LA (Figure 7). About 80% (52/65) of the elite temperate germplasms from China (Table 4). Four genes (*ZmCLA4*, *lg1*, *lg2* and *ZmTAC1*) have been shown to regulate LA, and a lot of maize inbred lines with favorable haplotypes were selected by haplotype analysis (Figure 8). The results indicated that there was a trend that the more favorable alleles, the smaller the leaf angle. The germplasms carrying favorable haplotypes can be used to improve maize plant architecture to increase planting density and increase maize yield.

Although GWAS has been recognized as a powerful method for understanding the genetic basis of complex quantitative traits, meta-analysis is complementary to GWAS and can increase precision and accuracy of detected QTLs. Therefore, combining GWAS and meta-analysis, we screened a lot of potential targets/loci regulating LA in maize, and the candidate gene, *ZmULA1*, was predicted to play important roles in the regulation of LA. These results will provide reference for improving maize plant architecture.

## Data Availability

The datasets presented in this study can be found in online repositories. The names of the repository/repositories and accession number(s) can be found below: https://www.ebi.ac.uk/eva/, PRJEB56161.
