# Supplementary material for: Candidate loci for leaf angle in maize revealed by a combination of genome-wide association study and meta-analysis
Source: Front Genet. 2022 Nov 11;13:1004211. doi: 10.3389/fgene.2022.1004211 (PMC9691904; doi:10.3389/fgene.2022.1004211)
Supplement: Supplementary file 1 [file DataSheet1.ZIP › supplementary files/FigureS1-S6.docx]

**
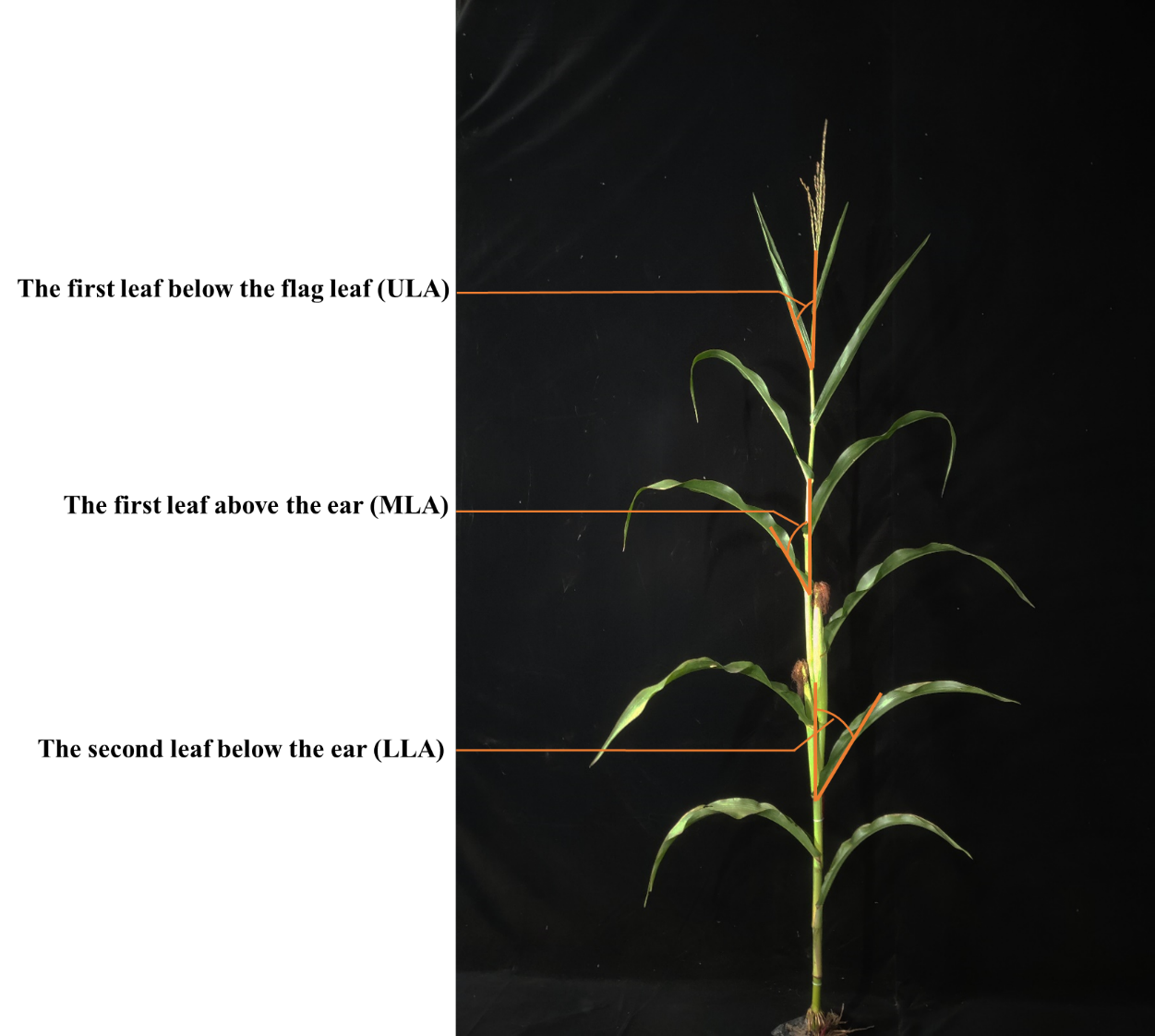
**

**Figure S1.** LA-related traits of maize plants.


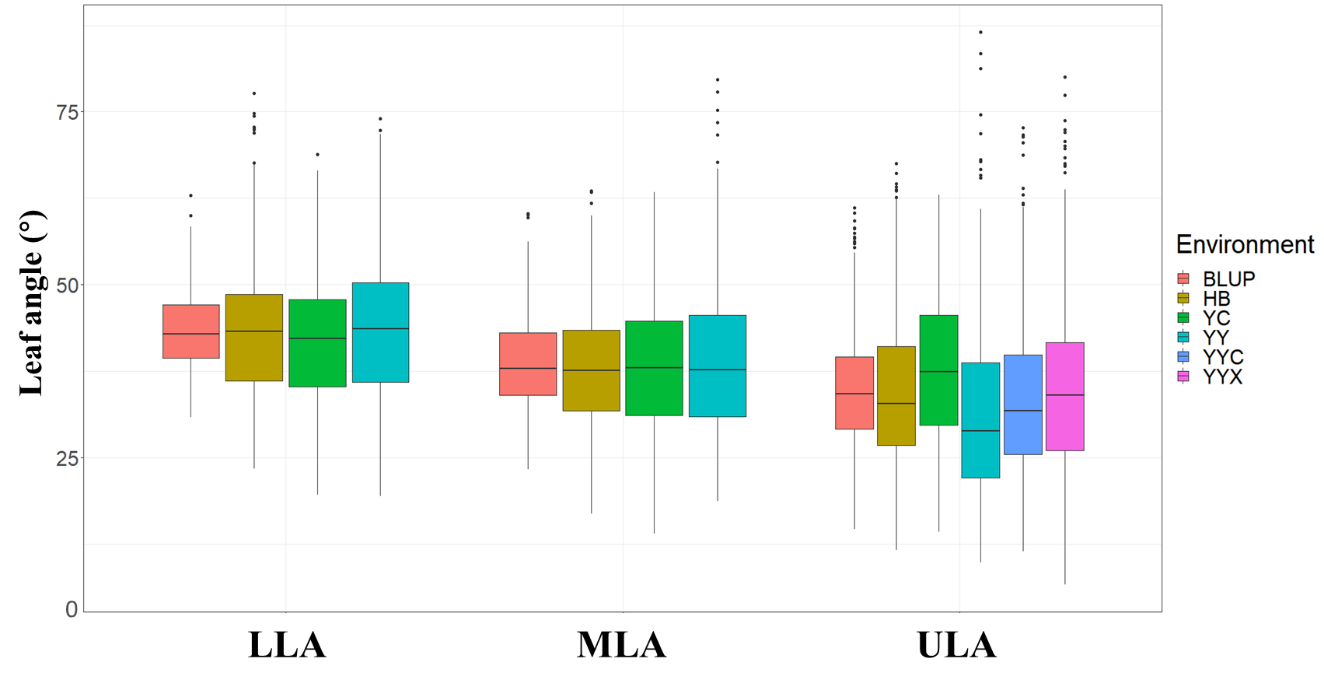


**Figure S2** Distribution of LA-related traits in different environments.


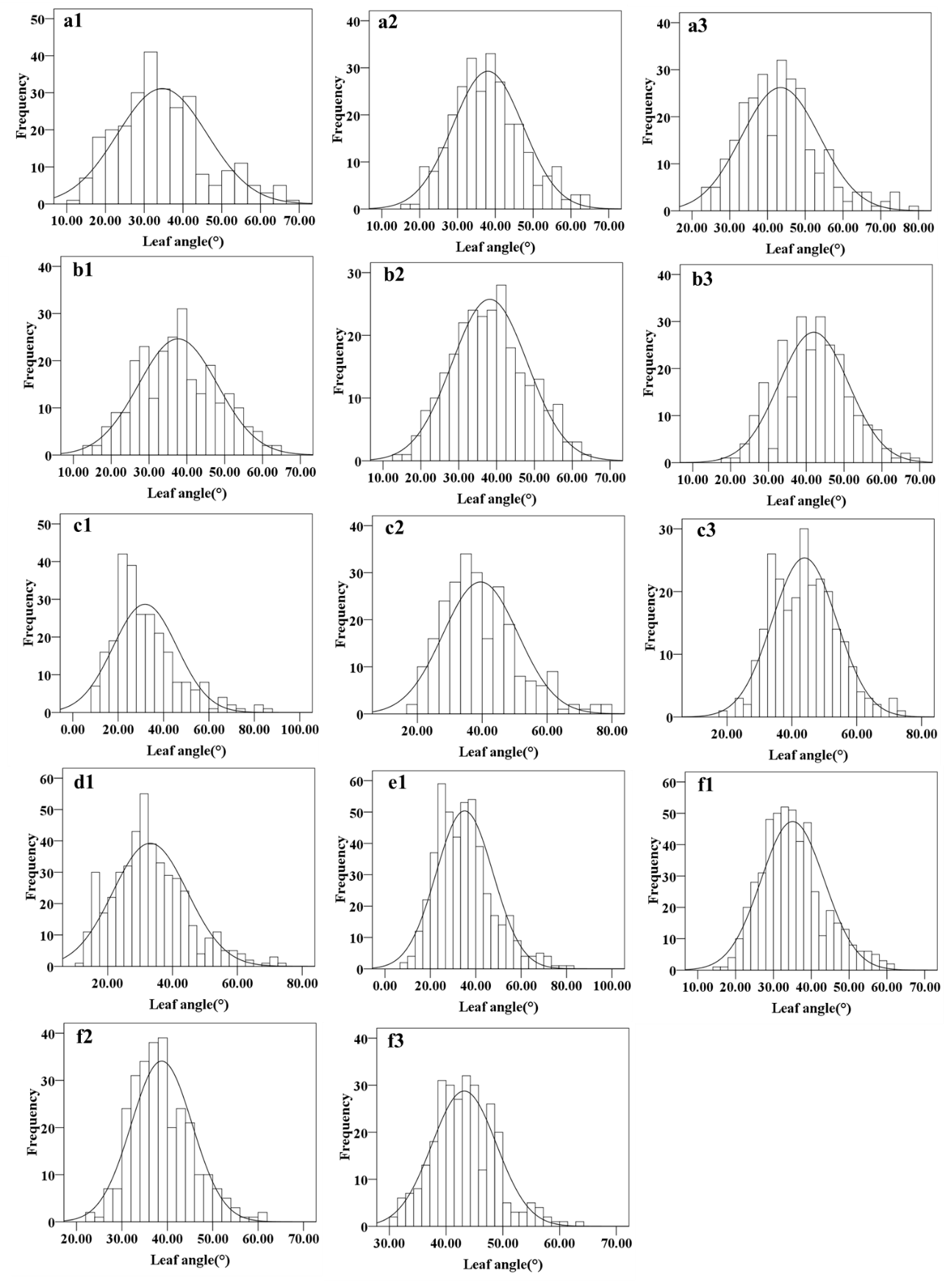


**Figure S3** Normal distribution of LA-related traits in different environments.

(a–f) represent HB, YC, YY, YYC, YYX, and BLUP, respectively; (1–3) represent ULA, MLA, and LLA, respectively.


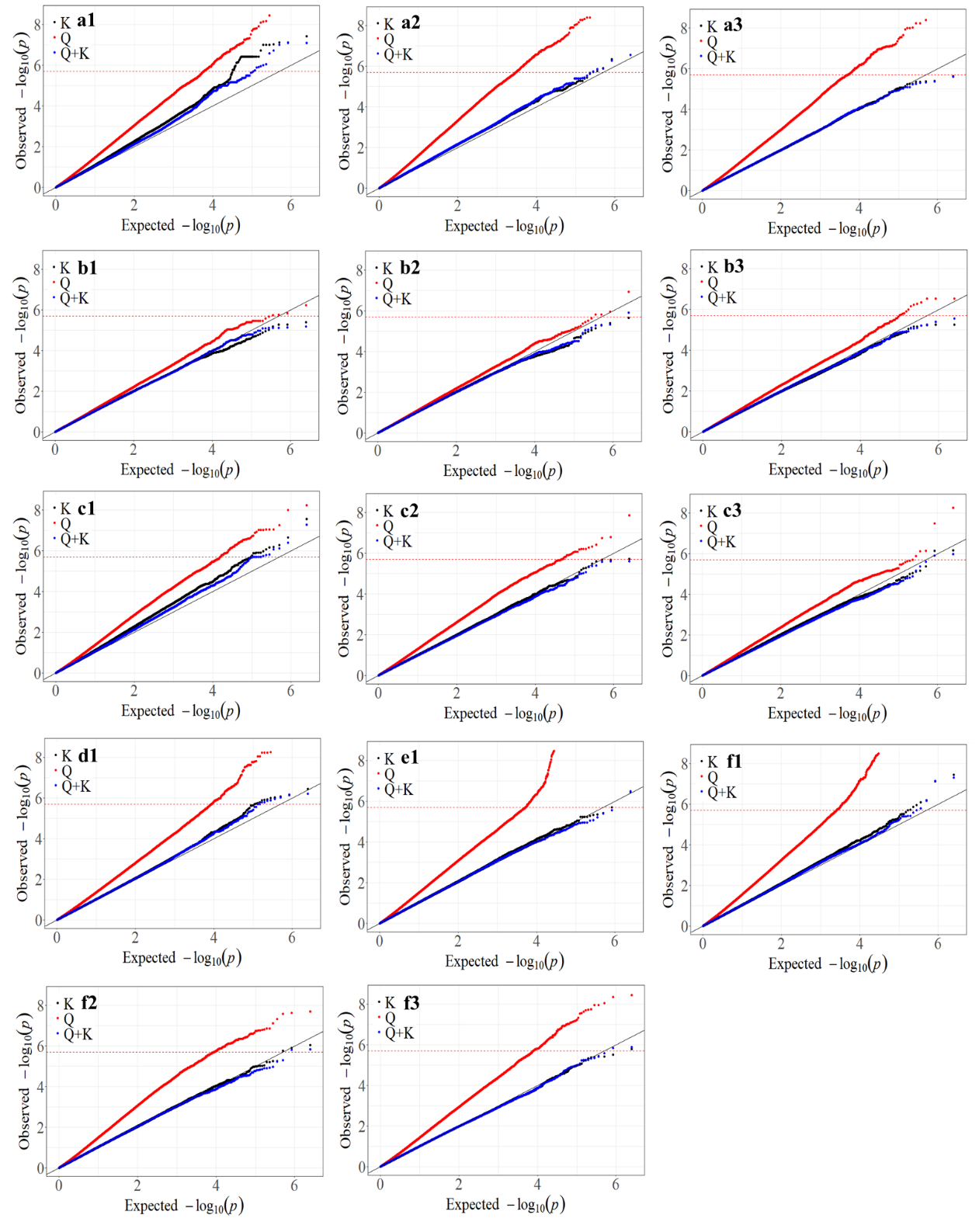


**Figure S4.** Quantile-Quantile (QQ) plots of LA-related traits using Q, K, and Q+K models.

(a–f) represent HB, YC, YY, YYC, YYX, and BLUP, respectively; (1–3) represent ULA, MLA, and LLA, respectively.


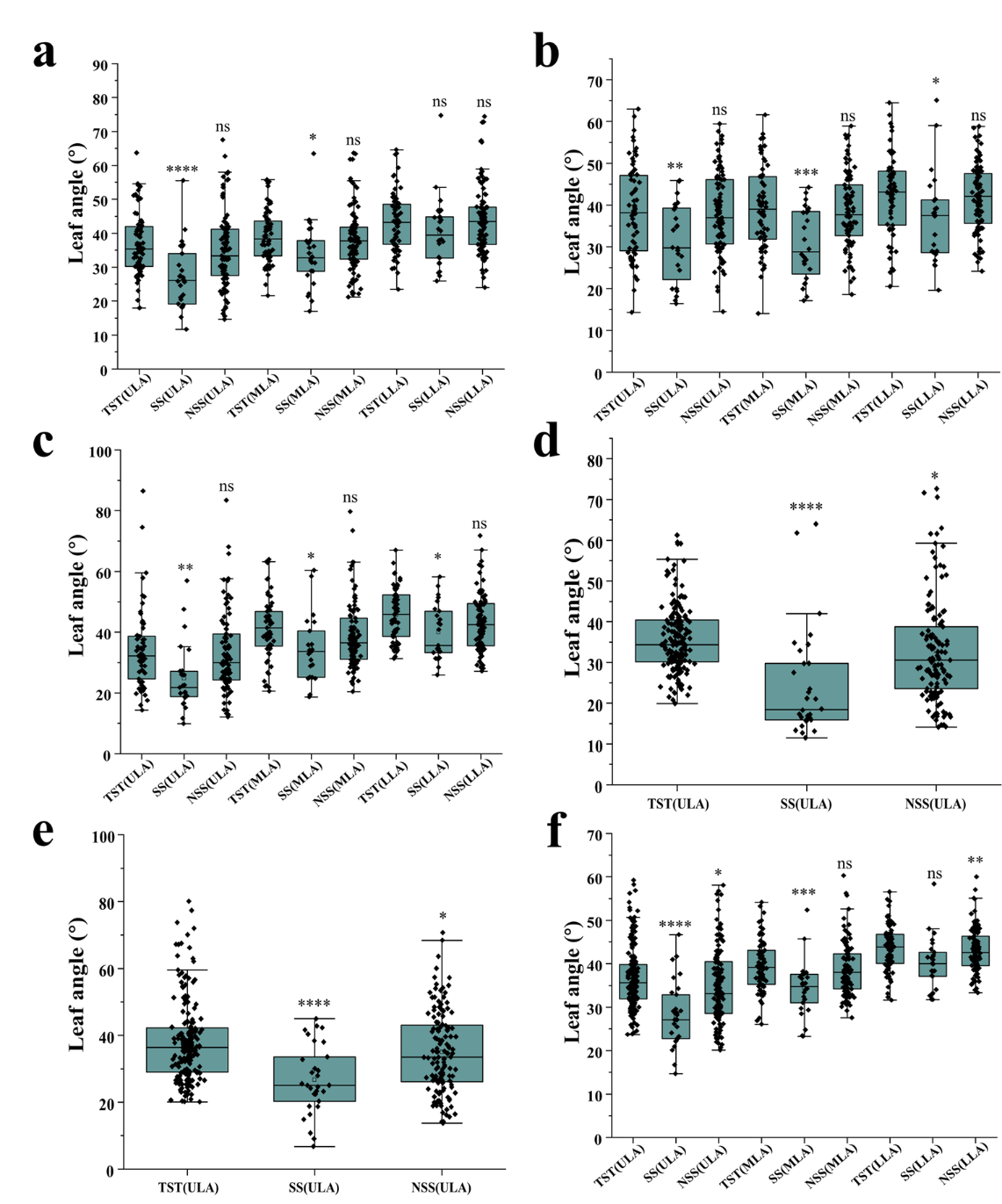


**Figure S5.** The distribution of the three LA-related traits in each sub-population across different locations.

a-f: HB, YC, YY, YYC, YYX, BLUP. TST, SS and NSS stand for Tropical/sub-tropical, Stiff Stalk and non-stiff stalk, respectively.

For each of the three LA related traits (ULA, MLA and LLA), the significance level was obtained by comparing SS or NSS with TST, respectively. *: P＜0.05, **: P＜0.01, ***: P＜0.001, ****: P＜0.0001, ns, not significant.


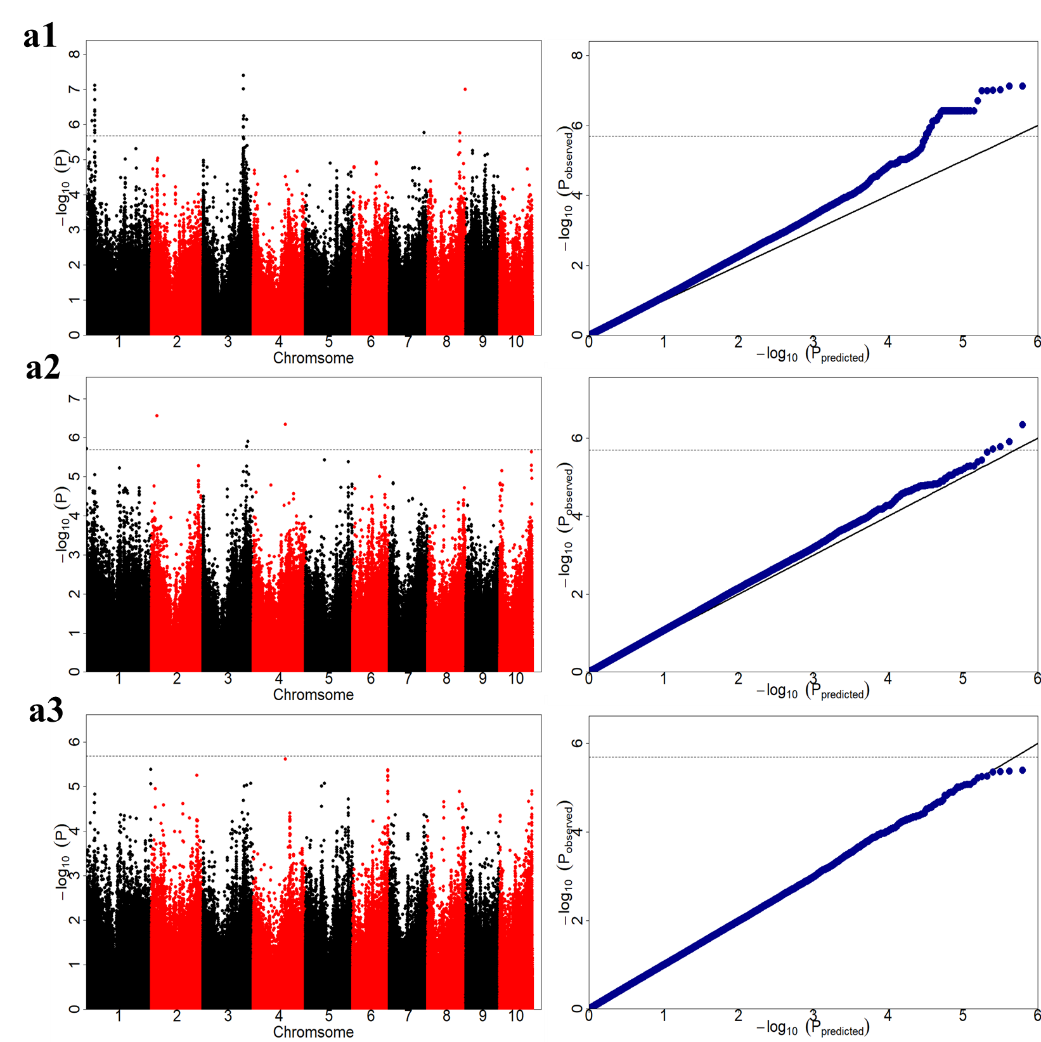


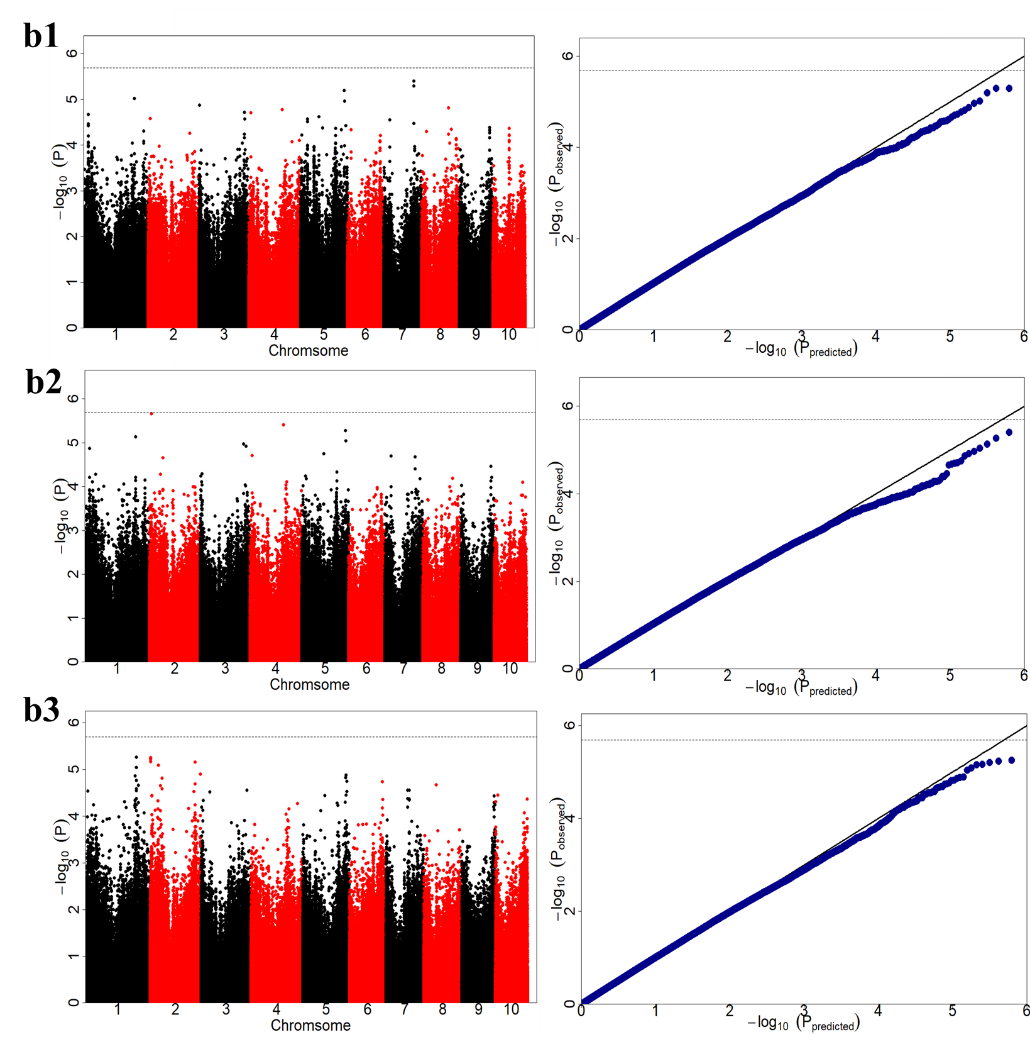


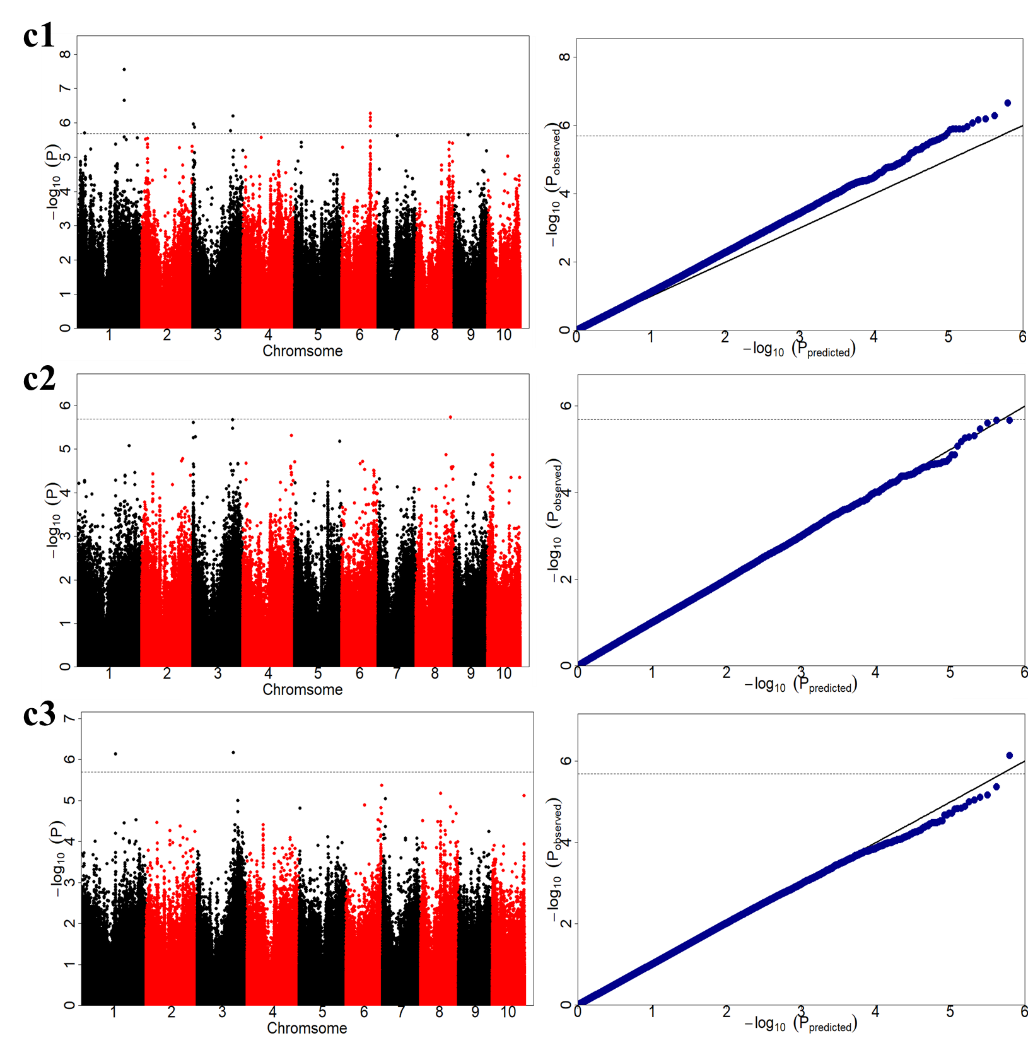


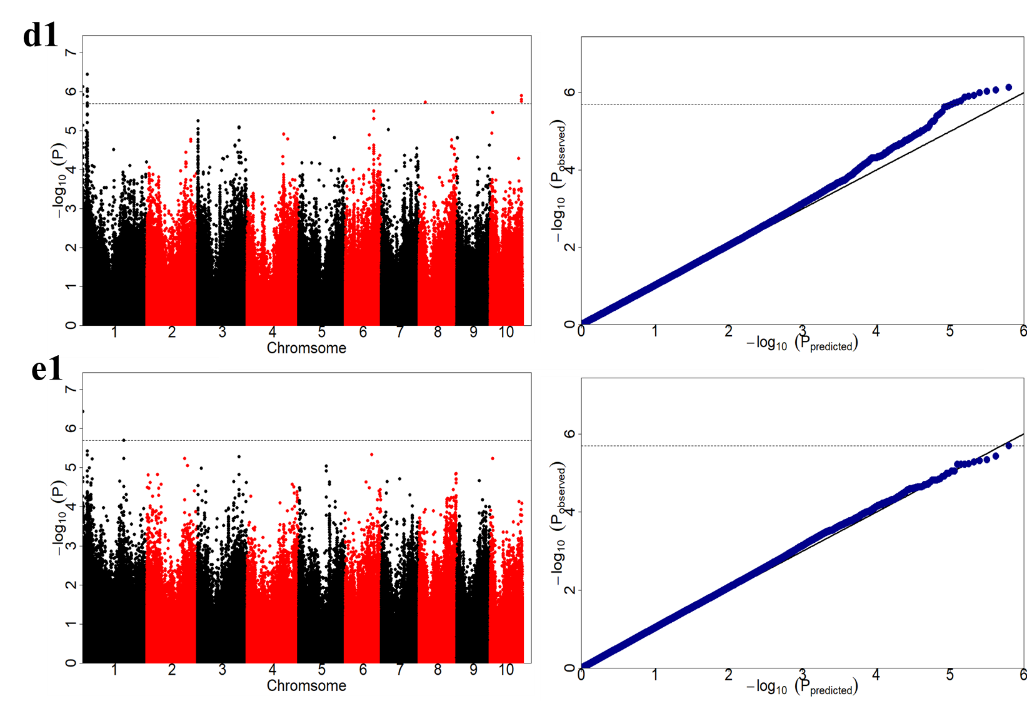


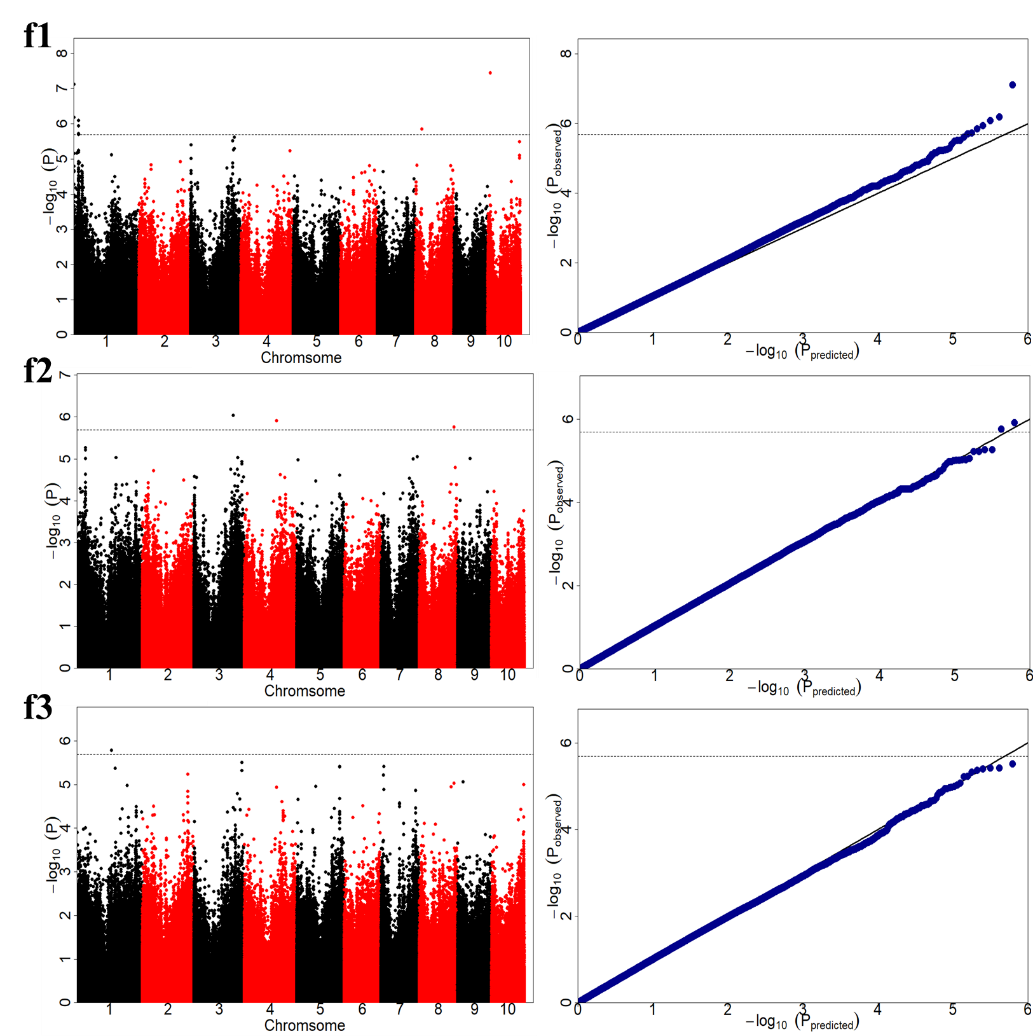


**Figure S6.** Manhattan plots and QQ plots of LA-related traits in different environments using the K model.

(a–f) represent HB, YC, YY, YYC, YYX, and BLUP, respectively; (1–3) represent ULA, MLA, and LLA, respectively.
